# New species of the Eastern Hemisphere genera *Afroheriades* and *Noteriades* (Hymenoptera, Megachilidae), with keys to species of the former

**DOI:** 10.3897/zookeys.159.2283

**Published:** 2011-12-23

**Authors:** Terry Griswold, Victor H. Gonzalez

**Affiliations:** 1 USDA-ARS Bee Biology & Systematics Laboratory, Utah State University, Logan, Utah 84322-5310, USA; 2 Present address: Division of Entomology, Natural History Museum, 1501 Crestline Drive – Suite 140, University of Kansas, Lawrence, Kansas 66045, USA

**Keywords:** Anthophila, Apoidea, Megachilinae, taxonomy

## Abstract

New species of the rarely encountered megachilid genera *Afroheriades* Peters from South Africa, *Afroheriades hyalinus* **sp. n.**, and *Noteriades* Cockerell from Myanmar and Thailand, *Noteriades jenniferae* **sp. n.** and *Noteriades spinosus* **sp. n.**, are described and illustrated. The species are described to make their names available for forthcoming publications on phylogenetic studies of the family Megachilidae. Taxonomic notes and a comparative diagnosis for each genus are presented. *Afroheriades hennigi* (Peters) and *Afroheriades reicherti* (Brauns) are new junior synonyms of *Afroheriades dolichocephalus* (Friese). A key to the known species of *Afroheriades* is provided.

## Introduction

The purpose of this paper is to describe three new species of rarely encountered Eastern Hemisphere megachilid bees, one species of *Afroheriades* Peters from South Africa, and two species of *Noteriades* Cockerell from Southeast Asia. Specimens of these undescribed species have been used in recent molecular analyses (i.e., Praz et al. 2008; Cardinal et al. 2010; Litman et al. 2011); herein we describe them to make their names available for use in forthcoming publications on the phylogeny of Megachilidae. New synonyms and revised comparative diagnoses for both genera are also presented.

*Afroheriades* and *Noteriades* consist of small heriadiform or hoplitiform bees (< 10 mm in body length) whose biologies are largely unknown (a species of *Noteriades* was observed entering a hole in an upright log). Each genus contains a small number of species, although several new species have been recognized by one of us (TG) in the past few years. *Afroheriades* is known only from South Africa, whereas *Noteriades* is found in tropical and subtropical regions of sub-Saharan Africa, India, and Southeast Asia (Table 1). Both taxa are currently included in the *Heriades* group of genera of the tribe Osmiini (Michener 2007) but little is yet known about their phylogenetic relationships; *Afroheriades* has been thought to be related to *Pseudoheriades* Peters while *Noteriades* has been considered related to *Heriades* Spinola. In fact, *Afroheriades* was originally described as a distinctive monotypic subgenus of *Pseudoheriades* (Peters 1970) whereas *Noteriades* was described as a subgenus of *Heriades* (Cockerell 1931).

**Table 1.** Summary of species currently included in *Noteriades*. Species distribution according to Griswold (1995), Ascher and Pickering (2011), and specimens deposited in the U.S. National Pollinating Insects Collection, Bee Biology and Systematics, Logan, Utah. * = Generic assignment unconfirmed.

**Species**	**Known distribution**
*Noteriades argentatus* (Gerstäcker, 1857)	Mozambique, Angola, Namibia, South Africa
*Noteriades bicornutus* (Friese, 1904)	Botswana, Congo, Zimbabwe, Zaire, Mozambique, South Africa
*Noteriades capensis* (Friese, 1922)	Zimbabwe, South Africa
*Noteriades chapini* (Cockerell, 1933)	Congo
*Noteriades clypeatus* (Friese, 1904)	South Africa
*Noteriades heterostictus* (Cockerell, 1936)	Zimbabwe
*Noteriades himalayensis* Gupta & Simlote, 1993*	India
*Noteriades infasciatus* Simlote & Gupta, 1993*	India
*Noteriades jenniferae* sp. n.	Thailand, Myanmar
*Noteriades pulchripes* (Cameron, 1897)	India, Myanmar
*Noteriades quinquecostatus* (Strand, 1912)	Zaire, Equatorial Guinea, Gabon, Central African Republic, Ivory Coast
*Noteriades spinosus* sp. n.	Thailand
*Noteriades striolatus* (Cameron, 1906)*	India
*Noteriades tergofasciatus* Simlote & Gupta, 1993*	India
*Noteriades tricarinatus* (Bingham, 1903)	Congo, Zaire, South Africa
*Noteriades zulu* (Strand, 1919)	South Africa

A recent comprehensive molecular analysis of the Osmiini (Praz et al. 2008) challenges these ideas regarding the taxonomy and relationships of *Afroheriades* and *Noteriades*. The molecular analysis supported the long suspected non-monophyly of Osmiini (e.g., Michener 2007), with *Afroheriades*, *Noteriades*, *Pseudoheriades*, and *Ochreriades* Mavromoustakis, excluded from the tribe (Praz et al. 2008). *Afroheriades* and *Pseudoheriades* formed a well-supported clade, but their phylogenetic position remained unclear; depending on the method employed in the analysis, they were either sister to the Anthidiini (parsimony analysis) or Megachilini + *Noteriades* (Bayesian analysis). *Noteriades* was consistently found to be the sister group of Megachilini (Praz et al. 2008). Such a relationship to Megachilini was first suggested by Griswold (1985) based on the presence of two apical spines on the outer surfaces of the fore and middle tibiae, and the modified hairs on the fifth sternum of the male. Except for the position of *Noteriades*, an on-going morphological phylogenetic analysis of Megachilidae (Gonzalez et al. in prep) is not concordant with the molecular analysis of Praz et al. (2008). In fact, *Afroheriades*, *Pseudoheriades*, and *Ochreriades* appear close to other “Osmiini” within a clade of most of the *Heriades* group (Gonzalez et al. in prep). Pending completion of that morphological analysis both genera are retained in the Osmiini.

## Material and methods

Morphological terminology follows that of Michener (2007). Photomicrographs were taken using a Keyence® VHX-500F Digital Imaging System. Measurements were made with an ocular micrometer attached to a Leica® MZ12 stereomicroscope. Measurements in descriptions are for the holotype, with values for paratypes in parentheses. The abbreviations F, S, and T are used for antennal flagellomere, and metasomal sternum and tergum, respectively.

Institutional acronyms used herein are:

AMNHAmerican Museum of Natural History, New York, NY, USA (J. Rozen, J. Ascher)

BBSLU.S. National Pollinating Insects Collection, Bee Biology and Systematics Laboratory, Utah State University, Logan, UT, USA

BPBMBernice P. Bishop Museum, Department of Entomology Collection, Honolulu, HI, USA (N. Evenhuis)

CASCalifornia Academy of Sciences, San Francisco, CA, USA (W. Pulawski, V. Lee)

CUICCornell University Insect Collection, Ithaca, NY, USA (B. Danforth)

MCSNMuseo Civico di Storia Naturale “Giacomo Doria”, Genova, Italy (R. Poggi)

SAMSouth Africa Museum, Cape Town, South Africa (V. Whitehead, M. Cochrane)

SANCSouth African National Collection of Insects, Pretoria, South Africa (C. Eardley)

TMPTransvaal Museum, Pretoria, South Africa (M. Scoble)

UCDCUniversity of California, R.M. Bohart Museum of Entomology, Davis, CA, USA (L. Kimsey)

ZMBMuseum für Naturkunde, Humbold-Universität zu Berlin, Berlin, Germany (F. Koch)

## Systematics

### Tribe Osmiini Newman

#### Genus Afroheriades

Peters, 1970

http://species-id.net/wiki/Afroheriades

Pseudoheriades  (*Afroheriades*) Peters, 1970: 157. Type species: *Pseudoheriades primus* Peters, 1970, by original designation.
Archeriades  Peters, 1978: 337. Type species: *Eriades larvatus* Friese, 1909, by original designation.

##### Diagnosis.

*Afroheriades* can be distinguished from all other Osmiini by the combination of posterolateral angle of scutum with marginal ridge non-carinate, with dense patch of long hairs laterally (Fig. 11) and T1 with juncture between anterior and dorsal faces not carinate. *Afroheriades* is morphologically similar to *Pseudoheriades* sharing a two-segmented maxillary palpus, female T6 with distinct apical hyaline flange, male T7 quadrately surrounded by T6, and male S3 with gradulus projecting into thin, basal hyaline lamella. In addition to the characters that distinguish it from all other Osmiini, *Afroheriades* differs from *Pseudoheriades* in: pronotal lobe and omaulus rounded; and male S3 without midapical spine. In *Pseudoheriades* the pronotal lobe and omaulus are distinctly lamellate; marginal ridge of posterolateral angle of scutum carinate, without dense patch of long hairs; T1 with distinct carina separating anterior and dorsal surfaces; and male S3 with midapical spine.

**Figures 1–10.**
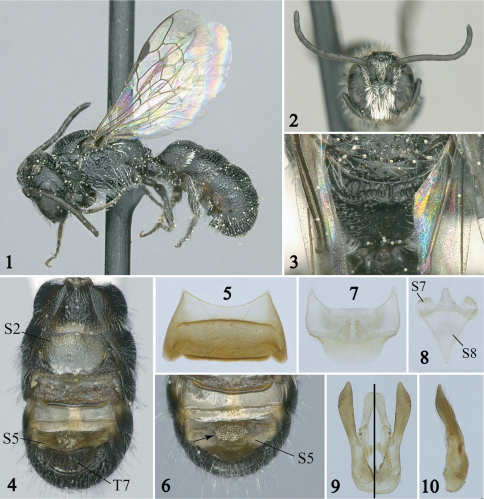
Male of *Afroheriades hyalinus*, sp. n. (paratypes except holotype in Fig. **1**) **1** Lateral habitus **2** Facial view **3** Metanotum and pitted propodeal base bounded posteriorly by a carina **4** Ventral view of metasoma; note the large hyaline lamella of S2 **5** S3 **6** Detail of S4 and S5 with arrow pointing to erect, oval tuft of bristles **7** S6 **8** S7 and S8 **9**, **10** Genital capsule in dorsal (left half), ventral (right half), and profile views.

##### Comments.

Two species groups can be recognized in *Afroheriades*: one includes a rather robust form with the basal area of propodeum well below the level of the scutellum [*Afroheriades primus* (Peters)]; the other includes the remaining species, which have a more elongate mesosoma and the base of the propodeum at the same plane as the scutellum. If a phylogenetic analysis shows that these two groups are natural, they could be subgenerically separated as *Afroheriades* s.str. and *Archeriades*, as suggested by Michener (2007). For now, we treat them as species groups. Below are the species currently recognized in *Afroheriades*; the synonyms presented here are based on the study of the types by the senior author. *Afroheriades capensis* Griswold, in Michener (2007: 452), is a *nomen nudum*.

##### *Afroheriades dolichocephalus* (Friese)

*Osmia dolichocephala* Friese, 1925: 505 (Holotype: AMNH; ♀, Cape Province, South Africa)

*Archeriades hennigi* Peters, 1978: 340 (Holotype: SAM; ♀, Cape Province, South Africa), new junior synonym.

*Eriades reicherti* Brauns, 1929: 140 (Lectotype: TMP; ♀, Cape Province, South Africa), new junior synonym.

##### *Afroheriades geminus* (Peters)

*Archeriades geminus* Peters, 1978: 339 (Holotype: SAM; ♀, Cape Province, South Africa)

##### *Afroheriades larvatus* (Friese)

*Eriades larvatus* Friese, 1909: 316 (Lectotype: ZMB; ♂, Cape Province, South Africa)

##### *Afroheriades primus* (Peters)

*Pseudoheriades primus* Peters, 1970: 157 (Holotype: SAM; ♂, Cape Province, South Africa)

#### Afroheriades hyalinus sp. n.

urn:lsid:zoobank.org:act:96738138-486E-431A-B1A8-3E23707C941D

http://species-id.net/wiki/Afroheriades_hyalinus

Figs 1 13

Afroheriades hyalinus Cardinal et al. (2010): Fig. S1, Table S1, S3 [supporting online information]

##### Holotype.

♂ (Fig. 1), South Africa, [Northern Cape]: Studer's Pass (km 23), -30.4288°, 18.0592°, 806 m, 17 Sep 2007, T.L. Griswold (SAM).

##### Paratypes.

10♂, 4♀, same data as holotype; 1♂, same data except *Lebeckia* sp.; 1♂ Concordia, 6 km N, -29.4859°, 17.9445°, 14 Sep 2007, T. L. Griswold; 10♂ 3♀, De Kruis, 14 km S, -30.503°, 18.1367°, 17 Sep 2007, T. L. Griswold; 2♀, Nieuwoudtville Falls, 6.5 km N of Nieuwandtville, -31.3197°, 19.1174°, 18 Sep 2007, T. L. Griswold; 1♀, Namaqualand, Bowesdorp, Sept 1941; 1♂, Garies, Namaqualand, June 1930. Western Cape: 1♂, Hoek se Berg, -32.1159°, 19.1734°, 20 Sep 2007, T. L. Griswold; 1♀, Pakhuis Pass, NE, -32.0922°, 19.0673°, 20 Sep 2007, T. L. Griswold; Worcester, IX-25-75, R.M. Bohart; 2♂, 1♀, Pakhuis Pass, 32.08S, 19.02E, 7.ix.1987, C. D. Eardley; 2♀, 5 km E Montagu, X 10 [19]75, R. M. Bohart; 2♀, 16 km E Clanwilliam, Pakhuis Pass, 700 m, 32°08.1'S, 18°59.7'E, 8 Sept 2001; 2♀, 7 km W Nieuwoudtville, 31°22.60'S, 19°01.16'E, 830m 9/X/2002, pantrap, F. D. Parker, M. E. Irwin; 1♀, Stradfontein, W of Muizenberg, 25 m, 34°04.86'S, 18°32.47'E, 7/X/2002, F. D. Parker, M. E. Irwin; 1♂, Cape Peninsula, Hout Bay, 18-IX-1967, E. S. Ross & A. R. Stephen (BBSL, CUIC, DZUP, SAM, SANC, SEMC, UCDC).

**Figures 11–13.**
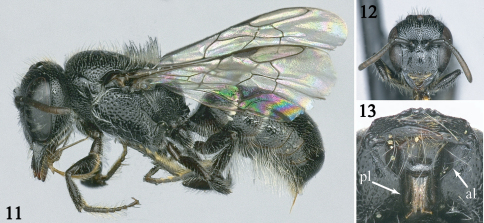
Female of *Afroheriades hyalinus*, sp. n. **11** Lateral habitus **12** Facial view **13** Ventral view of head showing anterior and posterior limbs of hypostomal carina. **al** = anterior limb or portion of carina curving towards posterior margin of mandibular socket; **pl** = posterior limb.

##### Diagnosis.

The male of *Afroheriades hyalinus* is unique among *Afroheriades* in the S5 with erect, oval tuft of bristles (Figs. 4, 6). In addition, males can be recognized by the combination of: antennal flagellum unmodified (Fig. 2); T7 without a lateral spine (Fig. 4); basal zone of the propodeum bounded posteriorly by a carina (Fig. 3); and anterior-facing surface of T1 with fine, sparse, punctures smaller than those on dorsal face. The female can be easily recognized by the combination of: head about as long as broad (Fig. 12); clypeus sparsely punctate, not produced ventrally; hypostomal carina with anterior limb (i.e., portion of carina curving towards posterior margin of mandibular socket) longer than the straight, posterior limb (Fig. 13); basal zone of propodeum horizontal, scarcely longer than length of metanotum, broader laterally than medially; and anterior face of T1 very finely, sparsely punctate.

##### Description.

*Male*: Body length 4.4 mm (3.7– 4.4 mm); forewing length 2.9 mm (2.6–2.9 mm). Head length equal to width (Fig. 2); ocellocular distance greater than ocellar diameter; ocelloccipital distance equal to ocellar diameter; compound eyes converging ventrally; antennal scape not enlarged, tapered; length of F2 twice length of F1, F1–F4 not expanded laterally, F2–F10 not roundly produced ventrally, F7 longer than wide; mouthparts in repose slightly exceeding fossa. Mesosoma elongate, cylindrical; pronotum roundly produced laterally; scutellum flat; metanotum horizontal in lateral view, midline length in dorsal view equal to length of basal zone of propodeum; propodeum with basal zone horizontal, surface pitted, bounded posteriorly by carina (Fig. 3). T6 roundly emarginate apically, apicolaterally rounded; apical margin of T7 convex, laterally without elongate spine (Fig. 4); S1 without subapical carina; S2 not thickened basally, with gradulus projecting into a thin, hyaline lamella apically reaching nearly to margin of segment (Fig. 4); S3 with margin transverse (Fig. 5); S6–S8, and genital capsule as in figures 7–10.

Integument smooth and shiny between punctures; punctation on interantennal area moderate, approximately one puncture width apart; on scutum moderate, approximately one puncture width apart; on disc of T3 sparse, more than one puncture width apart.

Body black except flagellomeres, tegula, legs dark brown. Wings hyaline with weak green and coppery highlights; veins, stigma, prestigma dark brown.

Pubescence white; dense, appressed, obscuring surface on clypeus, interantennal area, paraocular area; lower gena covered with sparse, loosely plumose hair; S2 with short, wedge-shaped fringe; S4 without median longitudinal hair tuft or carina; S5 with erect, oval tuft of bristles on disc (Fig. 6).

*Female*: As in male except: Body length 3.5–4.4 mm; forewing length 2.7–3.3 mm. Ocellocular distance 0.6–0.7 times interocellar distance; ocelloccipital distance 0.9–1.0 times interocellar distance; clypeus sparsely punctate, with margin truncate, crenulate; mandible tridentate, ventral margin not angled basally; gena ventrally without longitudinal carina, long curled hair, or ventral impunctate region; hypostomal carina higher posteriorly, curving towards posterior margin of mandibular socket, with anterior limb (curved portion) longer than posterior limb (straight portion), angle between limbs obtuse, area anterior to anterior limb of hypostomal carina raised (Fig. 13); labrum with medial area punctate, apical margin upturned; stipes without ventral fringe of long curled bristles. Dense pubescence restricted to paraocular area; hairs on fore tarsomeres simple, with tips wavy. S1 at most with preapical hump.

##### Etymology.

The specific epithet makes reference to the thin, hyaline lamella of S2 that projects from the gradulus distally nearly to the posterior margin of the sternum.

### Keys to species of *Afroheriades*

#### Males

**Table:** 

1	F2–F10 roundly produced ventrally	*Afroheriades geminus* Peters
–	F2–F10 not produced ventrally	2
2(1)	F1–F4 expanded laterally; basal zone of propodeum sloping posteriorly; mouthparts in repose not exceeding fossa; scutellum slightly convex	*Afroheriades primus* (Peters)
–	F1–F4 not expanded laterally; basal zone of propodeum horizontal; mouthparts in repose slightly exceeding fossa; scutellum flat	3
3(2)	Apical margin of T7 laterally with elongate spine; S4 with longitudinal carina medioapically; inner eye orbits parallel; ocelloccipital distance greater than ocellar diameter	*Afroheriades dolichocephalus* (Friese)
–	Apical margin of T7 laterally without elongate spine; S4 without longitudinal carina; inner eye orbits converging ventrally; ocelloccipital distance equal to ocellar diameter	4
4(3)	Basal zone of propodeum bounded posteriorly by carina (Fig. 3); F2 twice as long as F1	*Afroheriades hyalinus* sp. n.
–	Basal zone of propodeum not bounded posteriorly by carina; F2 no longer than F1	*Afroheriades larvatus* (Friese)

#### Females

**Table:** 

1	Gena ventrally with longitudinal carina fringed by long curled hair delimiting smooth impunctate ventral region; stipes with ventral fringe of long, curled bristles; clypeus closely punctate except sometimes along midline; area anterior to anterior limb of hypostomal carina not raised	*Afroheriades primus* (Peters)
–	Gena ventrally without longitudinal carina, long curled hair, or smooth impunctate ventral region; stipes without ventral fringe of long, curled bristles; clypeus sparsely punctate; area anterior to anterior limb of hypostomal carina raised	2
2(1)	Distance between middle and upper teeth of mandible at least two and a half times distance between lower and middle teeth; clypeal margin produced ventrally	*Afroheriades geminus* Peters
–	Distance between middle and upper teeth of mandible at most one and a half times distance between lower and middle teeth; clypeal margin not produced ventrally	3
3(2)	Hypostomal carina with obtuse angle, anterior limb equal in length to posterior limb (Fig. 13); inner eye orbits converging below; clypeal margin minutely crenulate; lateral ocelli approximately one ocellar diameter from occipital carina	4
–	Hypostomal carina with right angle, anterior limb shorter than posterior limb; inner eye orbits parallel; clypeal margin smooth; lateral ocelli approximately one ocellar diameter from occipital carina	*Afroheriades dolichocephalus* (Friese)
4(3)	Basal zone of propodeum almost twice as long as metanotum, not bounded posteriorly by a carina; anterior face of T1 virtually impunctate	*Afroheriades larvatus* (Friese)
–	Basal zone of propodeum scarcely longer than metanotum, bounded posteriorly by a carina (Fig. 3); anterior face of T1 distinctly, though finely, punctate	*Afroheriades hyalinus* sp. n.

### Genus Noteriades

Cockerell

http://species-id.net/wiki/Noteriades

Heriades  (*Noteriades*) Cockerell, 1931: 332. Type species: *Megachile tricarinata* Bingham, 1903, by original designation.
Noteriades : Michener 1938: 514; Griswold 1994: 26; Michener 2007: 475.

#### Diagnosis.

*Noteriades* can be easily separated from all other megachilid genera by the following combination of characters: small (4.5–10 mm), compact, hoplitiform bees with arolia present on all legs in both sexes; preoccipital carina complete; malar space linear; clypeus slightly projecting over clypeal-labral articulation; clypeus and usually supraclypeus with median longitudinal carina; pronotal lobe and omaulus carinate; scutellum carinate posteriorly; and propodeum vertical, without subhorizontal basal zone. *Female*. Mandible quadridentate, without differentiated cutting edges; T6 nearly vertical, except for apical hyaline flange. *Male*. Mandible bidentate; T1–T4 exposed dorsally, T5 and T6 curved ventrally, covering T7, S3 and remaining sterna; T6 without preapical carina; S1 subapically produced over its apical margin, forming a double carina; volsella distinct, with well-developed digitus and cuspis, and heavily sclerotized teeth resembling those of short-tongued bee families and within the Megachilidae, *Pararhophites* (Fideliinae).

#### Comments.

Griswold (1985; 1994) provided the first synoptic list of species of *Noteriades*, transferring most of them from *Heriades*. The species list presented in Table 1 is provisional; an on-going revision of the genus by the authors has revealed several undescribed species as well as new synonyms.

### Noteriades jenniferae sp. n.

urn:lsid:zoobank.org:act:F7D908F5-97FE-4DB0-89E8-7A254F4FA639

http://species-id.net/wiki/Noteriades_jenniferae

Figs 14 23 

#### Holotype.

**Figures 14–17.**
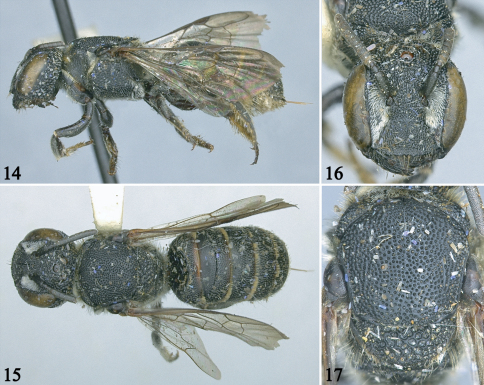
Female holotype of *Noteriades jenniferae*, sp. n. **14** Lateral habitus **15** Dorsal habitus **16** Facial view **17** Detail of mesosoma in dorsal view.

♀ (Figs. 14–17), Thailand: NW. Chiangmai Prov. Chiangdao; 450 m. IV-5-11-1958 / T. C. Maa, Collector, No. 321 (BPBM).

#### Paratypes.

6♂, same data as holotype, except one by “native collector” (BBSL, BPBM, CAS); 1♀ with the following label data: [Myanmar, see comments below] Carin Asciuii Chebá, 900-1000 m, L. Fea, IV-[18?]88/ Mus. Civ. Genova (MCSN).

#### Diagnosis.

This species is most similar to *Noteriades spinosus*, sharing with it the following characters: clypeus with median longitudinal carina extending onto supraclypeal area; juxtantennal carina absent (Fig. 16); and scutum with unmodified posterolateral angle, not spined or sharply angled, without tomentum obscuring integument (Fig. 17). It can be easily separated from *Noteriades spinosus* by: larger body size; head slightly broader than long (Fig. 16); and scutellum with posterior margin rounded, not projecting laterally into small, curved spine.

**Figures 18–23.**
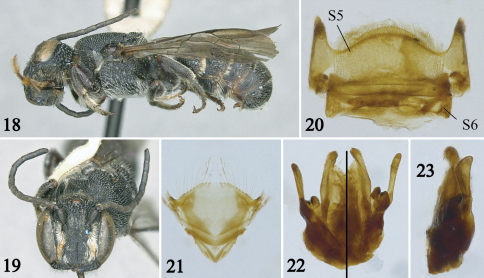
Male of *Noteriades jenniferae*, sp. n. **18** Lateral habitus **19** Facial view **20** S5 and S6 **21** S8 **22, 23** Genital capsule in dorsal (left half), ventral (right half), and profile views.

#### Description.

*Female*: Body length 9.0mm (8.0 mm); forewing length 5.6 mm (5.4 mm). Head slightly broader than long (Fig. 16); compound eyes subparallel, about 2.4 times longer than broad, 1.3 times wider than gena in profile; interantennal distance 1.9 times median ocellar diameter, 1.6 times antennocular distance; interocellar distance 1.4 times median ocellar diameter, shorter than ocellocular distance; ocelloccipital distance slightly longer than median ocellar diameter; clypeus about twice as broad as long, flat in profile, distal margin crenulate, median longitudinal carina distinct, extending onto supraclypeal area; juxtantennal carina absent; scape 2.4 times longer than wide, pedicel 1.3 times longer than broad, about twice as long as F1, F1 broader than long, about as long as F2, remaining flagellomeres progressively increasing in length, apical flagellomere longer than broad. Scutum with posterolateral angle not spined or sharply angled (Fig. 17); scutellum with posterior margin broadly rounded, without lateral spine.

Mandible and labrum with outer surfaces dull, minutely roughened, irregularly punctate, punctures slightly coarser on basal half of mandible, condylar and outer ridges smooth and shiny; clypeus with small (one-sixth to one-fifth times median ocellar diameter), contiguous punctures, smaller, denser on disc, smooth, shiny between punctures; supraclypeus with punctures as on sides of clypeus, punctures becoming larger (one-fourth times median ocellus diameter), slightly sparser on frons and superior paraocular area; inferior paraocular area with dense punctures smaller than on clypeus; vertex and gena with large (one-third to one-half times median ocellar diameter), coarse punctures, largest on gena; hypostomal area with punctures shallow, faint, separated by two puncture widths or less, integument smooth and shiny between punctures as on face. Mesosoma somewhat dull, imbricate between punctures except smooth and shiny on anterior surface of mesepisternum, metepisternum and most segments of legs; scutum uniformly punctate, punctures nearly contiguous, small (one-fourth to one-fifth times median ocellar diameter); axilla and scutellum with large (one-third to one-half times median ocellar diameter), coarse punctures as on gena (Figs. 15, 17); pronotum with punctures smaller than on scutum; anterior surface of mesepisternum minutely and densely punctate dorsally, impunctate ventrally; mesepisternum with large (one-fourth times median ocellar diameter) punctures separated by a puncture width or more, punctures becoming smaller and denser ventrally; metepisternum with small, contiguous punctures as on lateral surface of pronotum; metanotum and propodeal triangle shagreened, punctures faint, sparse on metanotum, remaining areas of propodeum with small, contiguous punctures; coxae finely, densely punctate, remaining areas of legs with large impunctate areas, punctures larger, sparser on hind legs. Metasomal terga and sterna smooth and shiny to finely imbricate, punctures smaller, denser than on scutum, punctures coarser, contiguous on distal terga (particularly T6), smaller, scattered on anterior face of T1.

Integument black throughout except tegula, legs, and metasomal sterna largely dark reddish brown. Wings light brownish with weak green or coppery highlights; veins, stigma, prestigma dark brown.

Pubescence in general short, sparse, white except yellowish on labrum, mandible, clypeal margin, and inner surfaces of tarsi; mandible and labrum with outer surfaces covered by dense, minute, erect hairs; paraocular area, dorsal surface of pronotum, pronotal lobe, lateral surfaces of coxae and propodeum with long, minutely branched hairs obscuring integument or nearly so; T1–T4 (also on T5 in paratype) with apical hair bands, medially interrupted on T1 and T2.

*Male*: As in female except longer and denser body pubescence, apical hair bands present on basal two terga only, coarser punctation, stronger median longitudinal carina of clypeus, and the following: Body length 8.0mm; forewing length 5.1 mm. Head 1.2 times broader than long (Fig. 19); compound eyes converging below, 1.4 times wider than gena in profile; interantennal distance about twice as long as median ocellar diameter and antennocular distance; interocellar distance 1.6 times median ocellar diameter, subequal to ocellocular distance; clypeus 1.6 times broader than long, with distal margin not as crenulate as in female; antennal flagellum long, surpassing distal margin of scutellum, scape robust, 1.6 times longer than wide, pedicel broader than long, longer than F1, F1 about one-third length of F2, remaining flagellomeres much longer than broad. S5, S6 and S8, and genital capsule as in figures 20–23.

#### Etymology.

This species is named after Mrs. Jennifer Lyman Strange, for her courage.

#### Comments.

The locality of the female paratype, Carin Chebá, is now known to be Karen Hills, Kayin (or Karan) State, Myanmar, a mountainous region about 40 km NE of Toungoo; the approximate coordinates are 19°13'N, 96°35'E (Jendek 2000: 189). The year this specimen was collected is not clear; it seems likely that it was 1888.

### Noteriades spinosus sp. n.

urn:lsid:zoobank.org:act:3396D3F8-3E2D-4F38-B8E5-CC5C2032688B

http://species-id.net/wiki/Noteriades_spinosus

Figs 24–27 

#### Holotype.

**Figures 24–27.**
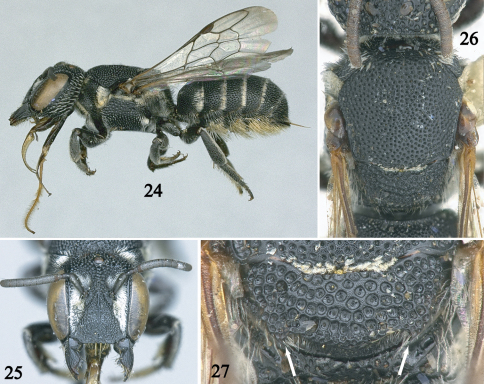
Female of *Noteriades spinosus*, sp. n. (paratypes except holotype in Fig. **24**) **24** Lateral habitus **25** Facial view **26** Detail of mesosoma in dorsal view **27** Detail of scutellum with arrows pointing to spines.

♀ (Fig. 24), Thailand: Chiangmai Prov [sic]., Doi Inthanon Nat. Park, Mae Ya waterfall / 28 March 1993, Leg. G. R. Ballmer (BBSL).

#### Paratypes.

(*n* = 9♀) Thailand: 7♀, Chiang Mai, 23 April 1988, W. J. Pulawski cllr; 2♀, NW Chiangmai Prov., Chiangdao, 450 m, iv-5-11-1958 [April 5-11, 1958] / T.C. Maa, Collector, No. 320, 336 (BBSL, BPBM, CAS).

#### Diagnosis.

This species is most similar to *Noteriades jenniferae* (see above). It can be easily separated from that species and all other *Noteriades* by the combination of: relatively small body size, head shape (about as long as broad, Fig. 25), and posterior margin of scutellum with small, curved spine laterally (Fig. 27).

#### Description.

*Female*. Body length 7.8 mm (7.1–7.8 mm); forewing length 4.8 mm (4.2–4.8 mm). Head about as long as broad (Fig. 25); compound eyes subparallel, about 2.4 times longer than broad, 1.3 times wider than gena in profile; interantennal distance about twice as long as median ocellar diameter, 1.5 times antennocular distance; interocellar distance 1.6 times median ocellar diameter, about as long as ocellocular distance; ocelloccipital distance 1.6 times median ocellar diameter; clypeus 1.7 times broader than long, flat in profile, distal margin crenulate, slightly projecting over clypeal-labral articulation, median longitudinal carina distinct on basal half, absent on distal half (thin but extending along clypeal length in some paratypes), extending onto supraclypeal area; juxtantennal carina absent; scape 2.6 times longer than wide, pedicel 1.4 times longer than broad, about twice as long as F1, F1 broader than long, about as long as F2, remaining flagellomeres progressively increasing in length, apical flagellomere longer than broad. Scutum with posterolateral angle not spined or sharply angled; scutellum with posterior margin rounded, laterally with small, curved spine (Fig. 27).

Mandible and labrum with outer surfaces dull, minutely roughened, irregularly punctate, punctures coarser on basal half of mandible, condylar and outer ridges smooth and shiny; clypeus with small (one-sixth to one-fifth times median ocellar diameter), rather shallow, contiguous punctures, smaller on disc, smooth, shiny between punctures; supraclypeus with coarser punctures than on clypeus, punctures becoming larger (one-third times median ocellus diameter) on frons, superior paraocular area, and vertex; inferior paraocular area with dense, shallow, smaller punctures than on clypeus; gena with large (one-half to two-thirds times median ocellar diameter), coarse punctures; hypostomal area with punctures small (about one-third times median ocellar diameter), shallower than on gena. Mesosoma weakly shiny, weakly imbricate between punctures except smooth and shiny on anterior surface of mesepisternum, metepisternum, propodeum and most segments of legs; scutum uniformly punctate (Fig. 26), punctures contiguous, small (one-fourth to one-third times median ocellar diameter); axilla as on scutum; scutellum with large (one-half times median ocellar diameter), coarse punctures; pronotum with smaller punctures than on scutum; anterior surface of mesepisternum minutely and densely punctate dorsally, impunctate ventrally; mesepisternum with small (one-third times median ocellar diameter) punctures separated by a puncture width or more, integument becoming shinier with smaller and denser punctures ventrally; metepisternum with coarse, contiguous punctures as on scutum; metanotum imbricate with sparse, coarse punctures; propodeal triangle smooth and shiny, impunctate, except imbricate and sparsely punctate basally; lateral and posterior surfaces of propodeum weakly shiny, with punctures contiguous, small (about-fifth times median ocellar diameter or less), faint; coxae finely, densely punctate, remaining areas of legs with large impunctate areas, punctures larger, sparser on hind legs. Metasomal terga and sterna weakly shiny, finely imbricate, punctures smaller, denser than on scutum, punctures coarser, contiguous on distal terga (particularly on T6), smaller, scattered on anterior face of T1.

Integument black throughout except tegula, legs, and metasomal sterna largely dark reddish brown. Wings light brownish with weak green or coppery highlights; veins, stigma, prestigma dark brown.

Pubescence in general short, sparse, white except yellowish on labrum, mandible, clypeal margin, and inner surfaces of tarsi; mandible and labrum with outer surfaces covered by dense, minute, erect hairs; paraocular area, dorsal surface of pronotum, pronotal lobe, mesepisternum near wing bases, lateral surfaces of coxae, and lateral surface of propodeum with long, minutely branched hairs obscuring integument or nearly so; T1–T4 with apical hair bands, medially interrupted on T1 and T2.

*Male*: Unknown.

#### Etymology.

The specific epithet makes reference to the curved posterolateral spine of the scutellum that distinguishes this species.

#### Comments.

Specimens of this species were used in the molecular analysis of the Osmiini by Praz et al. (2008).

## Supplementary Material

XML Treatment for Afroheriades

XML Treatment for Afroheriades hyalinus

XML Treatment for Noteriades

XML Treatment for Noteriades jenniferae

XML Treatment for Noteriades spinosus
